# Comparative Transcriptome Analysis of *Isoetes Sinensis* Under Terrestrial and Submerged Conditions

**DOI:** 10.1007/s11105-015-0906-6

**Published:** 2015-06-27

**Authors:** Tao Yang, Xing Liu

**Affiliations:** Laboratory of Plant Systematics and Evolutionary Biology, College of Life Science, Wuhan University, Wuhan, Hubei 430072 China

**Keywords:** *Isoetes* L., *Isoetes sinensis*, Transcriptome, Submerged condition, Terrestrial condition, Abiotic stress

## Abstract

*Isoetes* L. is an ancient genus of heterosporous lycopsids with a unique phylogenetic position. Repeated adaptations to environmental changes over time have contributed to occupying a variety of niches in *Isoetes*. However, we know little about how they adapt to the environmental changes, and the sequence resources are very limited in public databases. *Isoetes sinensis* is an amphibious plant in this genus, alternating frequently between terrestrial and aquatic environments. In this study, *I. sinensis* was applied to investigate the adaptations under terrestrial (TC) and submerged (ST) conditions using Illumina RNA-sequencing technology. Approximately 87 million high-quality reads were yielded and assembled into 31,619 unigenes with an average length of 1618 bp. Overall, 28,208 unigenes were annotated against the National Center of Biotechnology Information (NCBI), Non-redundant (Nr), Cluster of Orthologous Groups (COG), Gene Ontology (GO), and Kyoto Encyclopedia of Genes and Genomes (KEGG) databases. Moreover, we identified 1740 differentially expressed genes with 1146 up-regulated and 594 down-regulated genes under TC. GO annotation revealed that stress-relevant categories were remarkably enriched, and KEGG enrichment analysis showed that the phytohormone signalings and carbohydrate metabolism were significantly influenced. Furthermore, a total of 1646 transcription factors (TF) were identified and classified into 54 TF families; among them, 180 TFs were dynamic between terrestrial and submerged conditions. This study is the first report for *Isoetes* to generate numerous sequences and establish general understandings about the adaptations in the changing environments. The dataset provides a foundation for novel gene discoveries, comparative genomics, functional genomics, and phylogenetics in *Isoetes*.

**Electronic supplementary material:**

The online version of this article (doi:10.1007/s11105-015-0906-6) contains supplementary material, which is available to authorized users.

## Background

*Isoetes* L. (family Isoetaceae, quillworts) is an ancient genus of heterosporous lycopsids with a unique position in plant evolution (Pigg 1992). Phylogenetic analyses show that *Isoetes* is one of the earliest basal vascular plants, which can date back to the Devonian Period (Foster and Gifford 1959; Pigg 2001). *Isoetes* has approximately 200 species characterized by a strong reduced plant body (Li et al. 2012; Pigg 1992; Pigg 2001). Furthermore, this genus is the only survival of ancient taxa as the closest relatives of the famous tree lycopods (Hoot and Taylor 2001; Pigg 1992; Pigg 2001). Since the Paleozoic Era, repeated adaptations to environmental changes over time have contributed *Isoetes* to range from evergreen aquatics to ephemeral terrestrials. To date, the genus occupies a variety of niches, including oligotrophic soft-water lakes, higher altitude wetlands, seasonal pools, and intermittent streams (Keeley 1987; Pigg 1992; Pigg 2001; Taylor and Hickey 1992). The habitat preferences reflect past adaptations to the environmental changes, and play an essential role in the phylogenetic evolution of *Isoetes* (Keeley 1987; Pigg 1992; Taylor and Hickey 1992). Nonetheless, we know little about how *Isoetes* adapt to the environmental changes. Moreover, sequence resources are very limited in the National Center of Biotechnology Information (NCBI) database.

*Isoetes sinensis* Palmer is an allotetraploid plant (2*n* = 4*x* = 44) distributed in East Asia (Liu et al. 2001). Recent phylogenetic research has indicated that *I. sinensis* probably derives from hybridization between the diploid *I. yunguiensis* (2*n* = 22) and *I. taiwanensis* (2*n* = 22) (Liu et al. 2004). In addition, *I. sinensis* is a typical amphibious plant, which grows mainly in seasonal pools or intermittent streams and alternates frequently between terrestrial and aquatic environments (Keeley 1987; Wang et al. 2006). Thus, *I. sinensis* is a good model for *Isoetes* to research the adaptations to the environmental changes.

High-throughput sequencing technology makes it possible for non-model plants to produce multiple sequences at a relatively low cost. De novo assembly has been proven to be an ideal method for short reads in non-model organisms (Haas et al. 2013). In addition, RNA-sequencing approach has become a popular method to discover novel genes and explored different expression profiles under various conditions. In the last few years, there were substantial reports in RNA-sequencing datasets and expression profiles from lower to higher species (Der et al. 2011; Shih et al. 2014). In this study, our main objectives are to obtain numerous sequence resources and establish general understandings about how the *Isoetes* species adapt to the environmental changes. cDNA libraries from juvenile leaves of *I. sinensis* under terrestrial (TC) and submerged (SC) conditions were sequenced on an Illumina Hiseq 2000 platform, respectively. The dataset yielded multiple sequence resources and plotted a dynamic expression profile under TC and SC. GO annotation revealed that stress-relevant categories were remarkably enriched, and KEGG enrichment analysis showed that the phytohormone signalings and carbohydrate metabolism were significantly influenced. We further analyzed transcription factors (TF) to reveal their potential functions in *I. sinensis* for adapting to the environmental changes.

## Materials and Methods

### Plant Materials and Growth Conditions

*I. sinensis* was collected from Xinan River with a fluctuated water level in Zhejiang Province, China (29° 28′ N; 119° 14′ E). The plants were cultivated in the greenhouse of Wuhan University. All materials initially bred under SC with approximately 60 ml tap water for a month. Some experimental materials still maintained under SC about 4 cm below the surface of “artificial floodwater”, which were defined as control groups in this study. The others were transferred to TC for an additional month, and all leaves were entirely exposed to the air. Owing to the important roles of plant leaves in adapting to the changing environments (Baerenfaller et al. 2012), juvenile leaves under TC and SC were immediately harvested, frozen in liquid nitrogen, and stored at −80 °C, respectively.

### RNA Isolation and Sequencing

Total RNA was isolated using Trizol (Invitrogen Inc., USA), and the residual DNA was removed using RNase-free DNase I (Takara, Da Lian, China) according to the manufactures’ protocols. The RNA quantity was checked using ND-1000 Nanodrop Spectrophotometer (Thermo Scientific, DE, USA), and the quality was verified using 2100 Bioanalyzer RNA Nanochip (Aligene, CA, USA). Ten micrograms of verified total RNA was pooled in an equal amount from three independent extractions into a combined sample. The DNA-free mRNA was captured by magnetic oligo(dT) beads, fragmented to a size of 200 bp, and then synthesized into the first strand cDNA with random hexamer primers. The second strands were further synthesized using RNase H (New England Biolabs Inc., Ipswich, MA, USA) and DNA polymerase (Invitrogen, Carlsbad, CA, USA). We further repaired the end fragments and ligated them with sequencing adaptors. Suitable fragment ranges for PCR application (200 ± 25 bp) were selected by agarose gel electrophoresis and purified using a QIAquick PCR extraction kit (Qiagen, USA). The constructed cDNA libraries were sequenced on an Illumina Hiseq 2000 platform following the manufacturers’ instructions (Illumina Inc., San Diego, CA, USA).

### De Novo Assembly and Functional Analysis of Transcriptome Sequencing Results

The raw reads initially performed a stringent quality control analysis using FastQC (Andrews 2010). The base quality thresholds contained removing adaptors, sequence-specific bias, polymerase chain reaction artifacts, ambiguous nucleotide reads with N, and low quality bases with average Phred scores less than 20. After filtering and trimming, all clean reads were merged and further de novo assembled into unigenes using Trinity program, setting k-mers length to 25 (Grabherr et al. 2011). Then all unigenes were aligned using BLASTx against the non-redundant (Nr) database with an *E* value cutoff of e^−05^. To annotate the unigenes which failed to be aligned to the Nr database, we further applied GetORF software to predict their orientations and underlying protein coding regions (Rice et al. 2000). All assembled sequences were further searched using BLASTx against the Gene Ontology (GO), Cluster of Orthologous Groups (COG), and Kyoto Encyclopedia of Genes and Genomes (KEGG) databases with an *E* value cutoff of e^−05^. Blast2GO was used to obtain GO annotations, and WEGO database was applied to give a broad overview of the annotations regarding biological process (BP), molecular function (MF), and cellular component (CC) (Ye et al. 2006). COG database was used to represent a phylogenetic lineage of the *I. sinensis* transcriptome. Moreover, KEGG database was employed to explore putative pathways using KEGG automatic annotation service (KAAS) with the bi-directional best hit method (Kanehisa 2002).

### Identification and Functional Characterization of Differentially Expressed Genes

To investigate dynamic expression profiles under TC and SC, the present frequency of each unigene was calculated and normalized into reads per kilobase per million mapped reads (RPKM) values (Mortazavi et al. 2008). The expression fold changes were calculated as the log_2_ ratio of the two RPKM values under TC and SC. False positive and negative errors were analyzed by calculating the false discovery rate (FDR) values, which also applied to adjust *p* values using R program. FDR significant scores ≤0.001 and |log_2_ (RPKM_TC/SC_)| ≥1 were used as the thresholds to identify DEGs. In addition, all DEGs were further used for GO annotations and KEGG enrichment analysis. GO Slim was performed to map the GO annotations to a plant GO Slim file using the Fisher’s tests with the cutoffs as FDR adjusted *p* values less than 0.01. We also used a hypergeometric test to assess significant enrichment levels by setting *p* values less than 0.05.

### Identification and Analysis of TFs

To identify putative TFs in the transcriptome, we downloaded all well-known TF protein sequences from the Plant Transcriptional Factor (PlnTFDB) Database (Riaño-Pachón et al. 2007). All unigenes and DEGs were subjected to a local BLASTx homology search against the well-known TFs, respectively. The threshold of default parameters is an *E* value cutoff of e^−10^. The top hits were further extracted from the BLASTx results using an in-house Perl script, which were considered as the putative TFs in this dataset.

### Validation of the Transcriptomic Results Using Quantitative Real-Time PCR

To validate the reliability of the transcriptomic results, 27 DEGs were selected for qRT-PCR tests (Bustin et al. 2009). Total RNA from the juvenile leaves under TC and SC was extracted using RNAiso Plus (Takara, Japan) according to the manufacturers’ instructions. Total RNA of 1.5 μg was reverse-transcribed into single strand cDNA using Primerscript^TM^ One Step RT-PCR Kit Ver. 2 (Takara, Japan). Gene-specific primers were designed using a free online primer design tool (http://primer3plus.com/cgi-bin/dev/primer3plus.cgi). The qRT-PCR analysis was performed on a CFX96 real-time PCR system (Bio-Rad, Hercules, USA). The cDNA was diluted tenfold and amplified in a 25-μl solution, containing 12.5 μl 2× SYBR premix, 0.25 pmol forward and reverse primers, 2.5 μl diluted cDNA, and 7.0 μl sterile water. The PCR program was 95 °C for 3 min for the initial denaturation, followed by 40 cycles of 95 °C for 30 s, annealing temperatures for 15 s, and finally 72 °C for 30 s. Annealing temperatures and related information about the DEGs are listed in Table S1. Melting curves were plotted to determine the specificity at the end of the PCR cycling over the range 65–95 °C. Baseline and threshold cycle (Ct) were determined automatically by Bio-Rad CFX Manager 2.1 software. The relative expression levels were calculated using 2^−△△Ct^ method (Livak and Schmittgen 2001) and normalized to the geometric average of Ct values with *Actin* as an internal control gene. All experiments and analyses were conducted in triplicate.

## Results

### Sequencing and De Novo Assembly

To develop a general view of the *I. sinensis* transcriptome, cDNA libraries from the juvenile leaves under TC and SC were sequenced on an Illumina Hiseq 2000 platform, respectively. In total, we obtained 47,905,510 raw pair-end reads under TC and 52,709,149 under SC, respectively (Table S2). After filtering and trimming, approximately 87 million clean reads were generated, corresponding to 41,344,520 clean reads under TC and 45,530,550 under SC, respectively. All clean reads are deposited in the NCBI Short Read Archive database. The accession numbers are SRR1646513 under TC and SRR1648119 under SC. Then all clean reads were merged and de novo assembled into 31,619 unigenes with a mean length of 1618 bp and half of the assembled length (N50) of 2350 bp (Table S3). In addition, the size distribution of unigenes ranged from 200 to more than 5000 bp (Fig S1). A total of 78.3 % unigenes were larger than 500 bp, and 31.1 % were larger than 2000 bp.

### Annotation and Functional Characterization of *I. Sinensis* Transcriptome

**Fig. 1:**
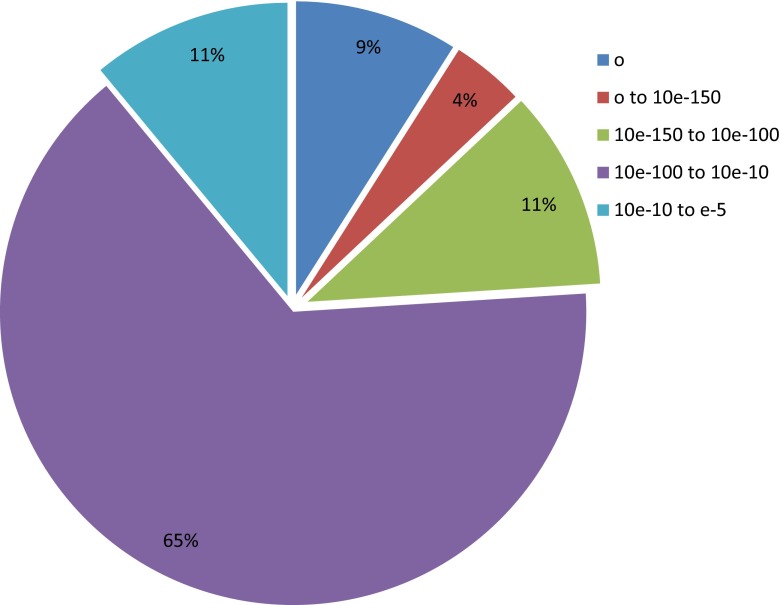
*E* value distribution of unigenes based on a BLASTx search against the Nr database

All assembled unigenes were further searched using BLASTx against the Nr, GO, COG, and KEGG databases (Table 1). A total of 28,208 (89.2 %) unigenes were similar to the known sequences in the Nr database with an *E* value cutoff of e^−05^ (Fig. 1). Overall, 15 % unigenes had strong similarities (*E* value < 10e^−100^) and 65 % presented moderate similarities (10e^−100^ < *E* value < 10e^−10^). In addition, 23,020 unigenes were assigned to at least one GO annotations using Blast2GO program. WEGO database was further used to map the annotations to particularly functional classification in terms of BP, MF, and CC. GO annotations of 52,492 were within 23 BP terms, 31,141 were within 14 MF terms, and 62,066 were within 16 CC terms (Fig S2). The most abundant term in BP was “metabolic process”, followed by “cellular process”, “response to stimulus”, and “biological regulation”. A large number of unigenes in MF were related to “binding” and “catalytic”. The commonest terms in CC were “cell” and “cell part”.

**Table 1:** Functional annotations of unigenes from BLASTx searches against the Nr, GO, COG, KEGG, and PlnTFDB databases

Databases	Numbers	Percentages
Nr	28,208	89.2
GO	23,020	72.8
COG	26,388	83.5
KEGG	7964	28.2
PlnTFDB	1646	5.8

**Fig. 2:**
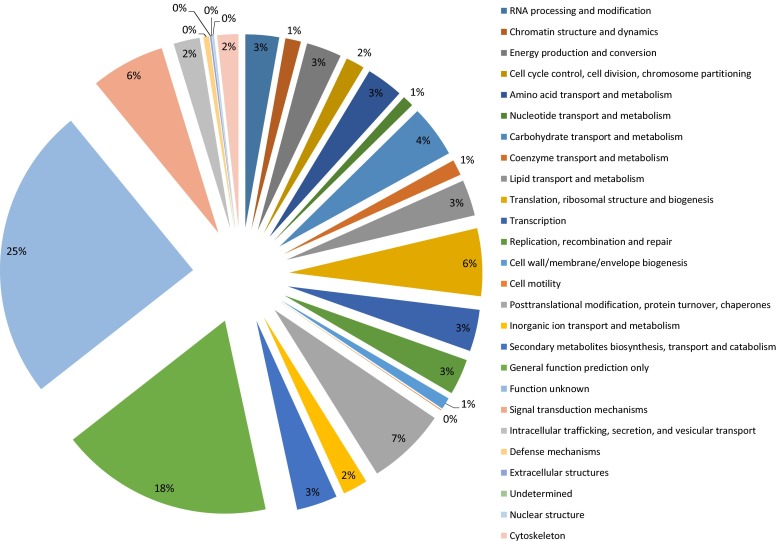
COG categories of assembled unigenes

All unigenes were further used to phylogenetically describe functional features using the COG database. A total of 26,388 unigenes were assigned to 25 COG classifications (Fig. 2). Ignoring “function unknown” category, the largest group was “general function prediction only” (18 %), followed by “posttranslational modification, protein turnover, chaperones” (7 %), and “signal transduction mechanisms” (6 %). The smallest groups were “nuclear structure” (0.18 %), “extracellular structures” (0.13 %), and “cell motility” (0.05 %).

To further elucidate the biological processes, the annotated unigenes were mapped into the reference pathways against the KEGG database. Unigenes of 7964 were annotated into 126 biological pathways in the KEGG database (Table S4). Unigenes related to “metabolic pathways” (1743 unigenes) were the largest, followed by “biosynthesis of secondary metabolites” (913 unigenes), “ribosome” (341 unigenes), and “plant hormone signal transduction” (255 unigenes).

### Identification and Functional Analysis of DEGs

**Fig. 3:**
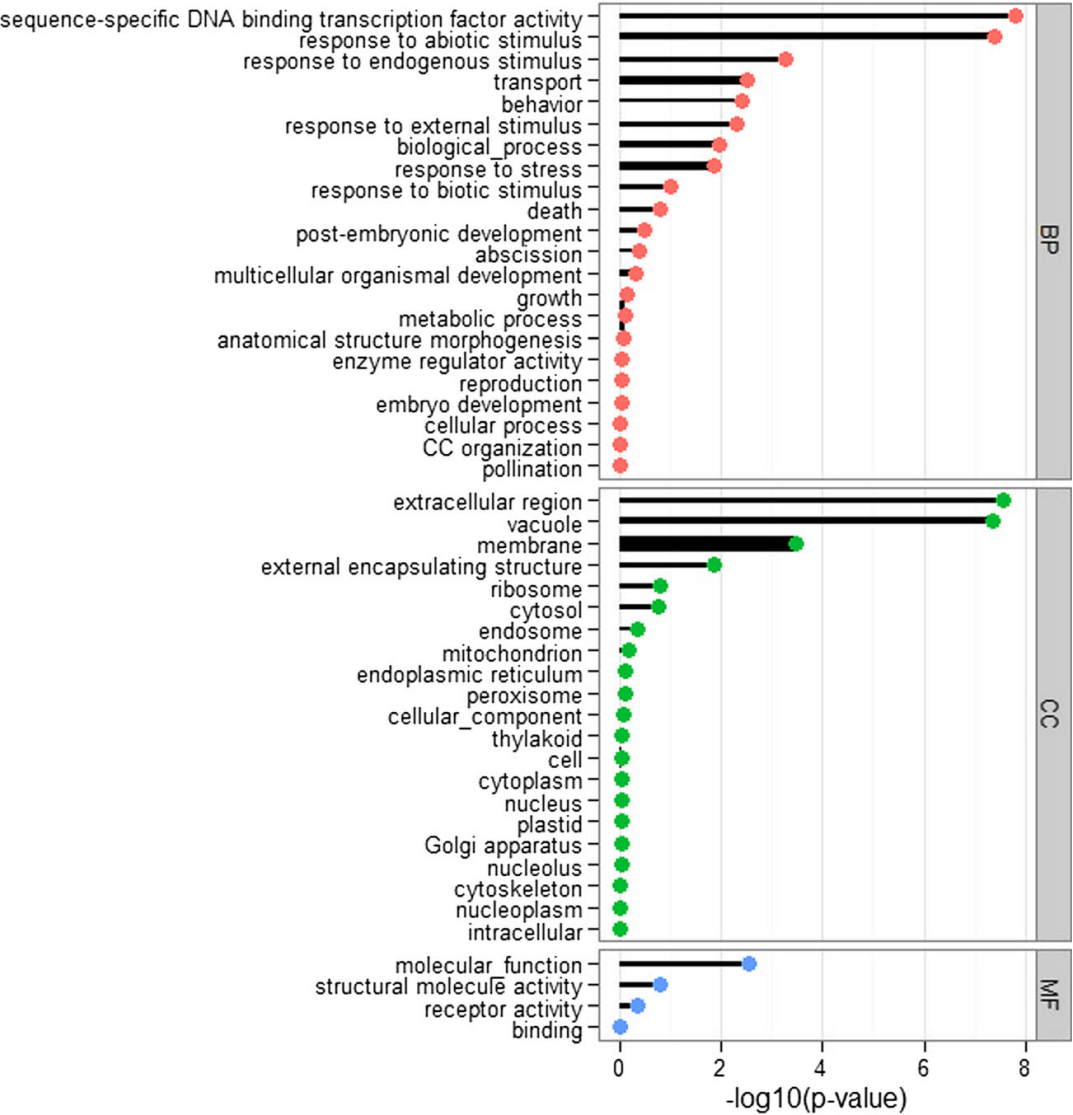
GO Slim functional classifications of DEGs. DEGs associate with GO annotations are represented regarding biological process (*BP*), cellular component (*CC*), and molecular function (*MF*). *X axis* indicates the functional significance of the enriched terms. *Y axis* describes the related GO terms. The *width of the lines* is the level of enriched numbers in each GO term, suggesting that the *wider lines* share more DEGs numbers in each term

We determined 1740 DEGs with the thresholds of FDR value ≤0.001 and |log_2_ (RPKM_TC/SC_)| ≥1. Comparing with SC, a total of 1146 DEGs were up-expressed and 594 were down-expressed under TC. To systematically characterize *I. sinensis* in response to the environmental changes, all DEGs were further used to investigate the GO annotations and KEGG enrichment pathways. We also applied a plant GO Slim file to give a broad overview of the GO annotations using Fisher’s tests with the cutoffs as *p* values less than 0.01. Of the 1740 DEGs, 1374 were assigned to 9184 GO annotations using Blast2GO. Moreover, 960 DEGs were significantly categorized to 4 MF terms, 3160 were within 21 CC terms, and 3035 were within 22 BP terms (Fig. 3). Large numbers of DEGs were significantly enriched in functional terms, such as “sequence-specific DNA binding transcription factor activity”, “response to abiotic stimulus”, “response to endogenous stimulus”, “response to stress”, “extracellular regions”, “vacuole”, and “membrane”.

**Fig. 4:**
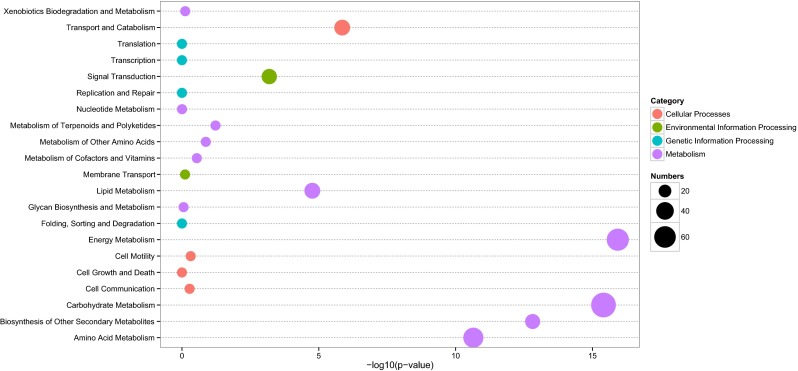
KEGG pathway assignments of DEGs. KEGG pathways contain the cellular process (*red points*), environmental information processing (*green points*), genetic information processing (*blue points*), and metabolism (*purple points*) categories. *X axis* indicates the functional significance of the enriched terms. *Y axis* is the KEGG pathway terms. The *point sizes* describe the enriched numbers in each term

We used a hypergeometric test to assign the DEGs to terms in the KEGG database. Of the 1740 DEGs, 386 were assigned and grouped into 96 KEGG pathways (Table S5). A total of 76.94 % DEGs were related to “metabolism” category, followed by “environmental information processing” (15.80 %), and “cellular processes” (9.85 %) (Fig. 4). Furthermore, the DEGs in the “metabolism” category was mainly over-represented in “carbohydrate metabolism” (*p* value 3.95e^−16^), followed by “energy metabolism” (*p* value 1.19e^−16^), and “biosynthesis of other secondary metabolites” (*p* value 1.54e^−13^). In addition, phytohormone signalings were also markedly enriched in “environmental information processing”, including ABA, ethylene, jasmonic acid (JA), salicylic acid (SA), cytokinin, auxin, and gibberellin signaling pathways (Table 2).

**Table 2:** Information about phytohormone signaling pathways under terrestrial and submerged conditions

GeneID	Pathway	Annotation	RPKM (T)	RPKM (S)	-log_2_ fold change	*p* value
comp48729_c1_seq1	ABA	*ABF*	15.09	8.21	0.85	0.1742
comp51265_c0_seq1		*ABF*	40.45	14.40	1.46	0.0124
comp47239_c0_seq1		*PYL*	42.17	189.52	−2.20	0.0002
comp59632_c1_seq8	Ethylene	*CTR1*	2.37	9.06	−1.96	0.0021
comp53115_c1_seq2		*ETR*	13.58	6.40	1.06	0.1021
comp54747_c0_seq2		*EBF1/2*	165.81	34.87	2.22	0.0002
comp50190_c0_seq2	JA	*COI-1*	37.81	6.81	2.45	0.0001
comp46730_c0_seq1		*JAZ*	68.65	8.21	3.04	0.0000
comp48472_c0_seq2		*JAZ*	14.34	2.19	2.68	0.0007
comp56477_c2_seq8		*JAR1*	2.52	0.11	4.51	0.0002
comp52220_c0_seq2	SA	*NPR1*	2.84	0.53	2.40	0.0058
comp44025_c1_seq1		*PR1*	0.23	5.34	−4.57	0.0001
comp61194_c0_seq1		*PR1*	0.43	6.73	−4.00	0.0008
comp55638_c0_seq1		*TGA*	10.72	3.66	1.52	0.0143
comp47186_c0_seq1	Cytokinin	*AHK2_3_4*	9.46	67.91	−2.87	0.0000
comp60573_c0_seq18		*AHK2_3_5*	16.51	59.51	−1.88	0.0013
comp39440_c0_seq1		*AHP*	34.34	7.22	2.22	0.0005
comp49013_c0_seq1		*A-ARR*	50.94	12.54	1.99	0.0007
comp48142_c0_seq1	Auxin	*IAA*	34.60	76.08	−1.16	0.0429
comp54483_c1_seq1		*SAUR*	0.32	1.80	−2.53	0.0460
comp51132_c1_seq1		*AUX1*	28.64	46.13	−0.72	0.2170
comp51568_c0_seq6	Gibberellin	*GID1*	12.34	4.24	1.51	0.0191

Phytohormone signaling pathways are present in the dataset, including ABA, ethylene, JA, SA, cytokinin, auxin, and gibberellin

### Identification and Analysis of TFs

In this study, we identified 1646 putative TFs using a local BLASTx homology search against the PlnTFDB. The 1646 TFs were classified into 54 out of 58 TF families in this dataset (Table 1). The most abundant TF family was MYB-related proteins, followed by NAC, C2H2, bHLH, ERF, MYB, C3H, and WRKY protein families (Table 3). To describe the dynamic regulations for TFs under TC and SC, all DEGs were also aligned to identify dynamic TF families. A total of 180 TFs were observed with 134 up-regulated and 46 down-regulated TFs. Furthermore, the most enriched TF families in the up-regulated DEGs were NAC and MYB-related proteins, followed by WRKY, bHLH, MYB, and ERF protein families; whereas the most abundant TF family in the down-regulated DEGs was bHLH protein, followed by MYB, AP2, B3, ERF, and MYB-related proteins (Table 3).

**Table 3:** Statistics of total and differentially expressed transcription factors related to the most abundant 12 transcription factor families

TF families	All unigenes	Up regulated	Down regulated
MYB related	107	10	2
NAC	78	10	1
C2H2	73	7	1
bHLH	72	8	5
ERF	59	7	2
C3H	53	0	0
MYB	53	7	3
WRKY	51	8	0
E2F/DP	41	3	1
FAR1	39	1	0
AP2	21	0	3
B3	41	1	2
Totals	1646	134	46

In this dataset, 1646 transcription factors (TF) are identified, and a total of 180 TFs are dynamic expressions with 134 up-regulated TFs and 46 down-regulated TFs

### Validation of the Transcriptome Using qRT-PCR

**Fig. 5:**
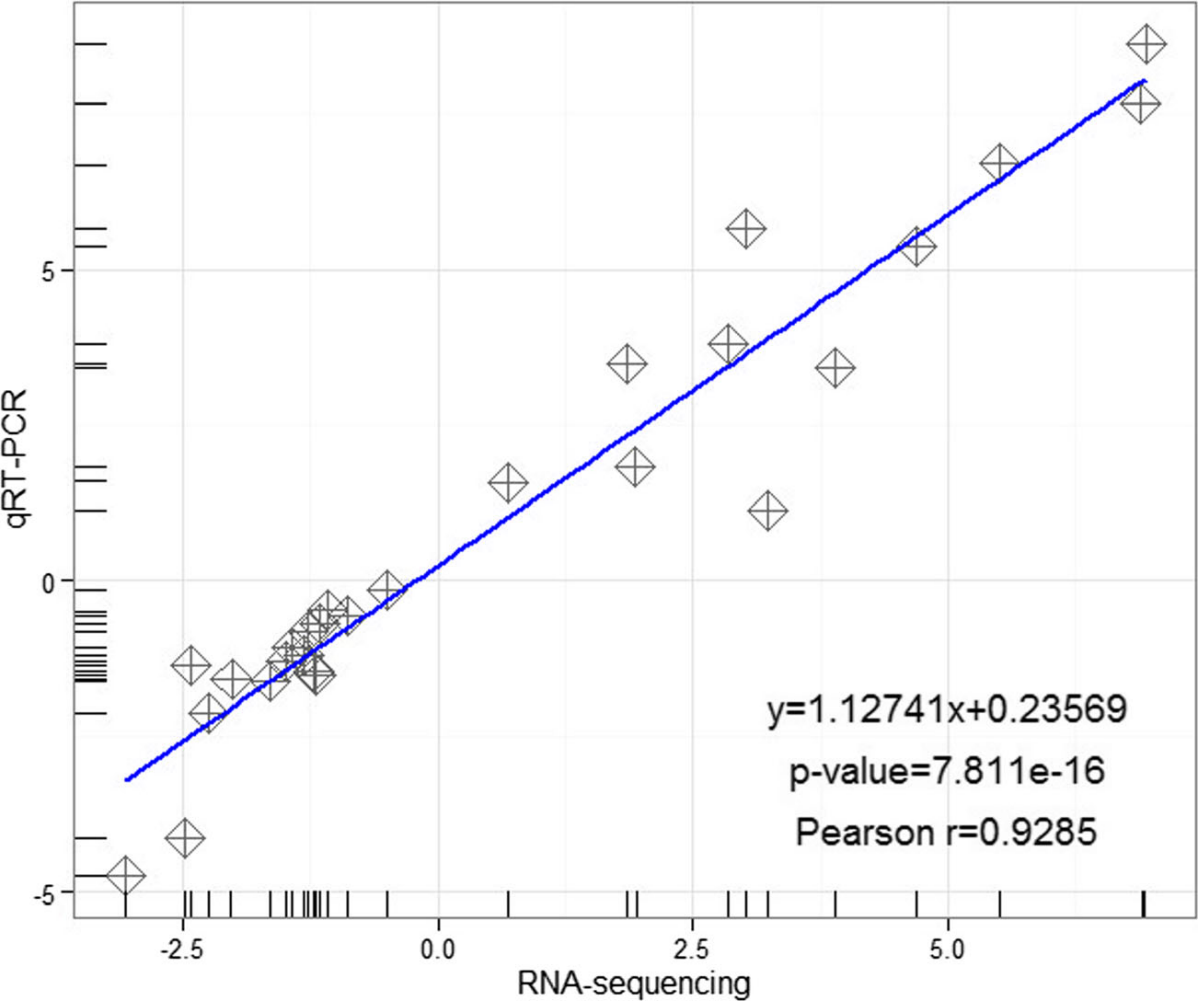
Correlation coefficient of fold changes between the transcriptome and qRT-PCR. The transcriptional levels of 27 DEGs are detected by qRT-PCR with three biological replications and technical replications. *Actin* is used as an internal control gene. A correlation coefficient of fold changes between transcriptome and qRT-PCR is calculated by R programs

To validate the transcriptomic results, we performed qRT-PCR analysis for 27 DEGs under TC and SC. Among the selected DEGs, 13 DEGs were randomly selected, and the others were related to phytohormone signaling pathways (Table S1). *Actin* was used as an internal control gene in this study. We used a Pearson correlation analysis to compare different expression levels between the transcriptome and qRT-PCR. There was a high correlation coefficient between the qRT-PCR and transcriptome results with Pearson *r* = 0.9285 (Fig. 5), further confirming that the transcriptomic results were acceptable.

## Discussions

RNA-sequencing approach is an effective alternative for non-model plants to obtain a large number of resources, and the technology is sensitive to detect dynamic ranges of the genes expression. In this study, we obtained a total of approximately 87 million high-quality reads using Illumina pair-end sequencing technology. This is the first report for *Isoetes* to produce substantial sequences using next-sequencing technology. The transcriptome provided reliable resources that more than 99 % reads passed the QC analysis. A high correlation coefficient was conducted between the results of qRT-PCR and transcriptome (*R*^2^ = 0.9256). In addition, the unigenes quality was favorable with commendable average length (1618 bp) and N50 length (2350 bp). To the best of our knowledge, average length and N50 of the unigenes were longer than the previous studies via comparing our transcriptome with previous de novo assembly sequencing data (Der et al. 2011; Gao et al. 2014; Shih et al. 2014).

Plants are sessile organisms, which need to face with environmental changes during the growth process. It is necessary for plants’ flexibly in response to different stresses in order to survive or reproduce offspring (Wang et al. 2003; Zeng et al. 2014). To date, the researches on environmental adaptations are mainly focused on higher plants or commercial crops (Hiz et al. 2014). GO annotation revealed that stress-relevant categories were remarkably enriched with the environmental changes. The analyses expand global understandings for adaptations to the environmental changes in basal plants, and indicate that stresses also play essential roles for *Isoetes* in the changing environments.

Hormones are important regulators for plants to adapt to environmental changes (Shan et al. 2013). Pathway enrichment analysis showed that phytohormone signalings were markedly enriched in “environmental information processing”, including ABA, ethylene, JA, SA, cytokinin, auxin, and gibberellin. ABA and ethylene are considered to be the most important phytohormones in response to abiotic stress (Peleg and Blumwald 2011). ABA is a central regulator in response to the environmental changes by triggering many stress-related genes and further increasing the associated tolerance (Chinnusamy et al. 2008). ABA regulations in the water-limited environments show that cellular dehydration increases trigger downstream targets, such as TFs, signaling factors, and the others (Yamaguchi-Shinozaki and Shinozaki 2006). ABA responsive element binding factor (*ABF*) is a key signaling factor belonging to the basic-region/leucine zipper TF family (Yoshida et al. 2010). In this dataset, two *ABF* homologues (comp48729_c1_seq1 and comp51265_c0_seq1) were identified and uniformly up-regulated under TC, suggesting that *ABF* played conserved roles in response to water-limited stress (Table 2). Ethylene signaling pathway has been well defined previously, and it is proven that ethylene interacts with ABA in response to stress (Stepanova and Alonso 2009). In ethylene signaling pathway, the *CTR1* (comp59632_c1_seq8), *ETR* (comp53115_c1_seq2), and *EBF1/2* (comp54747_c0_seq2) homologues were identified in the dataset. Furthermore, *EBF1/2* was significantly up-regulated, indicating that ethylene signaling was an important process for *I. sinensis* to adapt to the changing environments. Thus, ABA and ethylene interactions form a complex network in response to the environmental changes (Cramer et al. 2011).

JA and SA signaling pathways are considered to play important roles in response to biotic stress (Li et al. 2014). *COI-1*, *JAZ*, and *JAR1* homologues were identified and uniformly up-regulated under TC. The results suggested that *I. sinensis* probably had additional *JAZ* homologues, and they were negatively regulated by *COI-1* and *JAR1.* In SA signaling pathway, pathogenesis-related (*PR*)-1 is required to induce SA signaling and bound by *NPR1-TGA* complex (Dong 2004). Two *PR-1* homologues (comp44025_c1_seq1 and comp61194_c0_seq1) were identified and obviously down-regulated with 23.80 and 15.99-fold changes, respectively. The results indicated that SA signaling pathway played vital roles in response to stress, whereas more datasets should be supplied to expand global understandings for the SA regulations in *Isoetes*.

Carbohydrate metabolism is one of the most important primary processes and is associated with plants’ survival (McDowell 2011). Pathway enrichment analysis showed that 79 DEGs were significantly enriched in the “carbohydrate metabolism” category with 28 up-regulated and 51 down-regulated genes (Table S6), further confirming pivotal roles in survival from basal species to higher plants. Starch and sucrose metabolisms have been well investigated recently, which are dynamic with the effective synthesis and degradation (Tang et al. 2014; Yang et al. 2003). Furthermore, starch and sucrose act as potential signals interacting with stress to adapt to the environmental changes (Yang et al. 2003). Important genes related to starch and sucrose metabolisms were present in this dataset and influenced by the environmental changes, indicating that *I. sinensis* shared a complex regulatory network for starch and sucrose metabolisms to adapt to the environmental changes (Bianchi et al. 1991; Tang et al. 2014). Moreover, the concentrations of starch and sucrose are often consistent with the related genes’ regulations (McDowell 2011). Additional experiments need to demonstrate the consistency between genes’ expressions and concentrations of starch and sucrose. Glycolysis is also involved in adaptation to environmental stress, such as drought, cold, and osmotic stresses (Plaxton 1996). Furthermore, a general up-regulation of sucrose metabolism reveals enhanced glycolysis metabolisms in the level of transcription (Fernie et al. 2002; Fernie et al. 2004). In this study, DEGs related to glycolysis metabolisms were uniformly down-regulated, indicating that sucrose metabolism probably produced lots of energy under SC and it was mediated by glycolysis metabolism.

TFs are a group of proteins interacting with cis-regulatory elements of the target genes. An initial research states that TFs numbers are increasing from the algaes to dicots, such as 157 TFs in *Cyanidioschyzon merolae*, 1423 in *Physcomitrella patens*, and 2757 in *Arabidopsis thaliana* (Libault et al. 2009; Sharma et al. 2013). Furthermore, more complex organisms employ larger TFs to execute complicated regulations and metabolisms (Libault et al. 2009; Pérez-Rodríguez et al. 2009; Sharma et al. 2013). In this study, we identified 1646 putative TFs, further supporting the previous reports (Libault et al. 2009; Pigg 1992; Sharma et al. 2013). We further elucidated dynamic TF distributions with 134 up-regulated and 46 down-regulated TFs, which have been reported to play a pivotal role in response to the environmental changes (Hiz et al. 2014; Liu et al. 2014).

Multiple TF families are linked to abiotic or biotic stress, acting as an activator or repressor to regulate stress-induced changes, such as MYB, NAC, and WRKY proteins (Libault et al. 2009; Yu et al. 2012). Since the *COLORED1* locus was identified to encode a MYB domain protein, a large number of MYB-related TFs have been accumulated and found to be involved in plant-specific processes, such as primary metabolism, cell fate, and response to biotic and abiotic stresses (Chen et al. 2013; Dubos et al. 2010). NAC proteins are plant-specific TFs, which play key roles in plant development and response to biotic or abiotic stress. A recent research has stated that 151 *NAC* genes in rice and 117 in *Arabidopsis* are identified using a genome-wide analysis (Olsen et al. 2005). Moreover, phylogenetic analyses reveal that NAC TFs are divided into NAC-A, NAC-B, and NAC-C subgroups (Nakashima et al. 2012). The NAC-A and NAC-B subgroups probably emerge after the separations between lycophytes and other vascular plants (Nakashima et al. 2012). There were substantial NAC sequences in the dataset and they were dynamic in response to the environmental changes. As a group of the basal vascular plants, the phylogenetic position and divergence time in *Isoetes* are particularly important, needing to do more investigations in the future.

bHLH proteins are a group of global TFs found in animals and plants. Although many members in this family have not been elucidated, a few *bHLH* transcripts increase in drought, cold, and salt stresses, such as *AtAIB* and *bHLH-92* (Jiang et al. 2009; Li et al. 2007). WRKY proteins in *A. thaliana* are known to encode novel CaM-binding TFs (Rushton et al. 2010). WRKY TFs not only act as activators but also as repressors to play key roles in depression and repression of plant important processes (Park et al. 2005). Multiple previous reports have demonstrated that WRKY TFs participate in various abiotic or biotic stress responses (Rushton et al. 2010). Furthermore, single WRKY protein often involves in several stresses to regulate the transcriptional reprograms (Rushton et al. 2010). Eight WRKY TFs were identified and uniformly up-regulated under TC, indicating that WRKY TFs played a vital role in response to the water-limited environments (Chen et al. 2012).

Abbreviations

BP, biological process; CC, cellular component; DEGs, differentially expressed genes; GO, gene ontology; JA, jasmonic acid; KEGG, Kyoto Encyclopedia of Genes and Genomes; MF, molecular function; NCBI, National Center of Biotechnology Information; Nr, non-redundant; N50, half of the assembled length; qRT-PCR, quantitative real-time PCR; SA, salicylic acid; SC, submerged condition; TC, terrestrial condition; TF, transcription factor.

## Electronic supplementary material

Fig S1 Size distribution of unigenes. (GIF 59 kb)

High resolution image (TIFF 80 kb)

Fig S2 GO functional classifications of unigenes. (GIF 156 kb)

High resolution image (TIFF 349 kb)

Table S1 Primers information for quantitative real-time PCR in this study. (DOCX 18 kb)

Table S2 Statistics of the de novo assembly results. (DOCX 15 kb)

Table S3 Summary of unigenes in this study. (DOCX 15 kb)

Table S4 KEGG enrichment pathways of unigenes. (XLS 28 kb)

Table S5 KEGG enrichment pathways of differentially expressed genes. (XLS 26 kb)

Table S6 Statistical analysis of carbohydrate metabolism under terrestrial and submerged conditions. Seventy-nine DEGs are significantly enriched in “carbohydrate metabolism” with 28 up-regulated and 51 down-regulated genes. (DOCX 17 kb)
