# Supplementary figures and images for: Comparative Transcriptome Analysis of Isoetes Sinensis Under Terrestrial and Submerged Conditions
Source: Plant Mol Biol Report. 2015 Jun 27;34:136–45. doi: 10.1007/s11105-015-0906-6 (PMC4722078; doi:10.1007/s11105-015-0906-6)

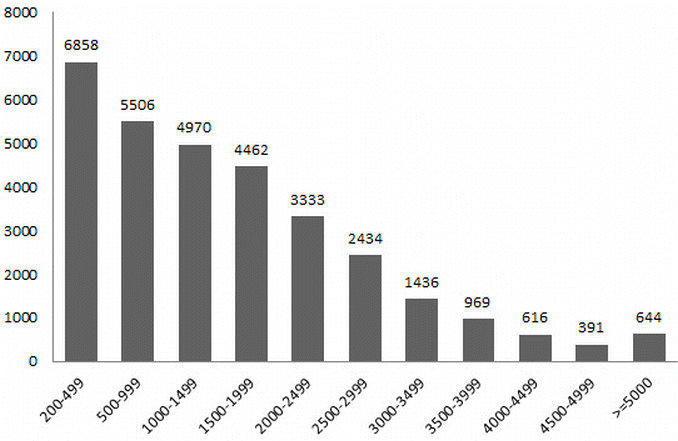

Supplement: Supplementary file 1 — Size distribution of unigenes. (GIF 59 kb) [file 11105_2015_906_Fig6_ESM.gif]

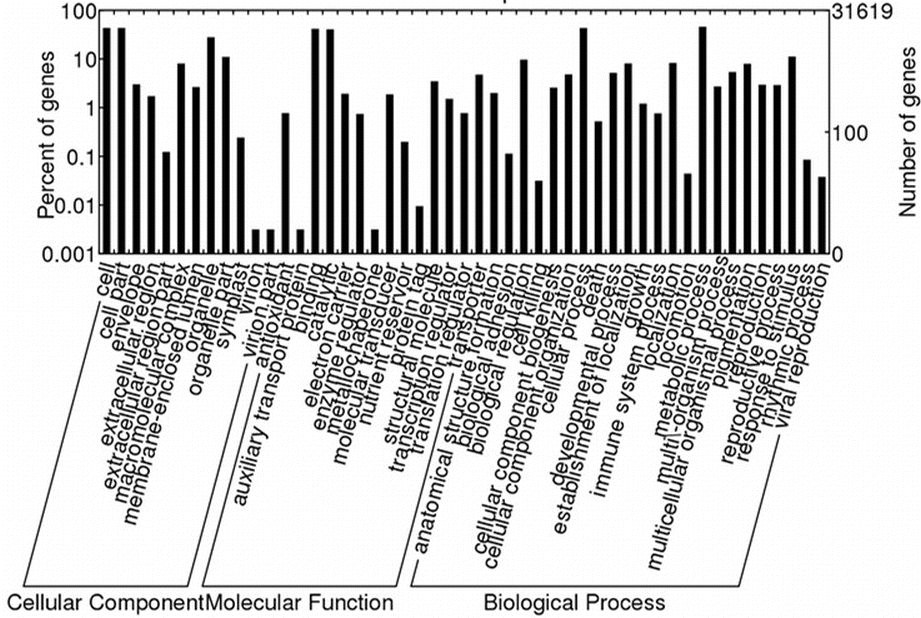

Supplement: Supplementary file 3 — GO functional classifications of unigenes. (GIF 156 kb) [file 11105_2015_906_Fig7_ESM.gif]
