# Supplementary material for: Comparative Transcriptome Analysis of Isoetes Sinensis Under Terrestrial and Submerged Conditions
Source: Plant Mol Biol Report. 2015 Jun 27;34:136–45. doi: 10.1007/s11105-015-0906-6 (PMC4722078; doi:10.1007/s11105-015-0906-6)
Supplement: Supplementary file 5 — Primers information for quantitative real-time PCR in this study. (DOCX 18 kb) [file 11105_2015_906_MOESM3_ESM.docx]

**Table S1** Primers information for quantitative real-time PCR in this study.

| Uingenes | Forward primers | Reverse primers | Temperatures | Annotations |
| --- | --- | --- | --- | --- |
| comp49013_c0_seq1 | GAGACTTCCTTGACCCTCTT | TGGCTGCACTTCCATAGC | 55 | A-ARR |
| comp51265_c0_seq1 | TTTTACCTGGTTCTCCCA | AACAACAGTTTCGGCATC | 54.5 | ABF |
| comp39440_c0_seq1 | CCTGGTTTTGTTGCTGAA | AGTCCATTGGCTCTTGAT | 54.5 | AHP |
| comp50190_c0_seq2 | ATTCCGTTGCCATTAGAT | GACTTCACTGTGCGCTTC | 55 | COI-1 |
| comp48472_c0_seq2 | TATGGCGGATCAGTTTGT | TCTGCTCGAATTATTTGCT | 55 | JAZ |
| comp52220_c0_seq2 | GTGGCTCCTGATGGTGTT | GCAGAAAGGTGGTGAAGAA | 55 | NPR1 |
| comp48729_c1_seq1 | CCACTGGGTATCTGGCAATC | CTTGAGGGATAAACCTTGGTAGTA | 56 | ABF |
| comp47186_c0_seq1 | AGCCCTTTCCGACTTCTT | TTACCGACCCTGCTGTTG | 56 | AHK234 |
| comp60573_c0_seq18 | TATGGTGGTACTGGGATAGG | TTTGTTGAGGCGTGCTTA | 55 | AHK235 |
| comp51132_c1_seq1 | AGAAGGGAAAGATGATTGC | TTCGAGATGTGGCTGTTAT | 54.5 | AUX1 |
| comp53115_c1_seq2 | TGGGTTCTGATTCTGTTCG | ATTGCCTACTTTCTTCTTCGT | 54.5 | ETR |
| comp55054_c0_seq1 | ATGGCTCTGATTATGTCC | TCATAATACGCAGTCTCCT | 56 | IAA |
| comp52020_c1_seq1 | AAGGGTGTCAGCAAGTGATT | TTATGTGGATGGGAGGGTT | 56 | TGA |
| comp58940_c1_seq4 | TTCCTATTACATTCCTTGGC | GCAACCTGTATGATCGAAAC | 56 | SAUR |
| comp49177_c0_seq1 | GCCAATGCTATGACCACCA | CCCCTGCCTTTTCCAATCT | 55 | Peroxidase |
| comp59083_c0_seq14 | ACAAGGGACTCCGAAGATACA | GGCAAGAACCAGCCTCTAATA | 55 | kinesin family member C3 (KIFC3) |
| comp59045_c0_seq1 | GCTCCTATTCATCCAAACACTG | GACCCTGAACCCACCACC | 55 | long-chain-alcohol oxidase FAO4A-like |
| comp49295_c0_seq3 | AGGAGTACACCCAGAACGAGC | TTACGCCAACCGACGATG | 55 | iron permease FTR1-like |
| comp57976_c0_seq8 | GGATGGTTACGGAGGAGCA | GGGAGGGACTGTTATTATTGGATA | 55 | de-epoxidase |
| comp54763_c0_seq1 | GTAGGGTGGGCGTCTTCG | CGGTCTTTACGGTGGCATCT | 60 | L-gulonolactone oxidase-like |
| comp56261_c0_seq2 | GGCTGCTATGCTTGGGTC | CGCACTCGAATCTCGTCC | 60 | ASPARTIC PROTEASE IN GUARD CELL 1-like |
| comp55857_c0_seq11 | ACAGAGCATCCGCAATACCA | TCATCGCCTTCCTTCAGC | 55 | DUF21 domain-containing protein AT4g33700-LIKE |
| comp53032_c0_seq1 | GGATGAATGGGCATACACG | TGTTCCTTCAGGGCTTCG | 55 | MYB128 |
| comp56980_c0_seq3 | GCGTTGAAGCGTAGTCGG | ATGCGGGTGAGCAGGATC | 55 | At1g04910-like |
| comp53589_c2_seq10 | TTCCCGACACTGCTACACC | CACAAATCCTCCTAATTCTAACCC | 60 | BTB/POZ domain-containing protein At5g03250-like |
| comp48729_c1_seq1 | TATGGACTGCGGAGGAGA | TAGCGGAACCACGATGAA | 60 | polyadenylation element-binding protein isoform |
| comp58205_c0_seq12 | TTCCTATTACATTCCTTGGC | GCAACCTGTATGATCGAAAC | 55 | auxin-induced protein 15A-like |
| comp40690_c2_seq1 | TCCTCTTCCAGCCTTCTTT | TTCCTCCACTGAGCACAATA | 54.5 to 60 | Actin |
