# Supplementary material for: Comparative Transcriptome Analysis of Isoetes Sinensis Under Terrestrial and Submerged Conditions
Source: Plant Mol Biol Report. 2015 Jun 27;34:136–45. doi: 10.1007/s11105-015-0906-6 (PMC4722078; doi:10.1007/s11105-015-0906-6)
Supplement: Supplementary file 6 — Statistics of the de novo assembly results. (DOCX 15 kb) [file 11105_2015_906_MOESM4_ESM.docx]

**Table S 2** Statistics of the de novo assembly results.

|  | TC | SC |
| --- | --- | --- |
| **Raw reads** | 47,905,510 | 52,709,149 |
| **Raw reads bases** | 9,581,102,000 | 10,541,829,800 |
| **Q20(%)** | 96.39 | 96.44 |
| **GC(%)** | 44.92 | 44.69 |
| **Clean reads** | 41,344,520 | 45,530,550 |
| **Clean reads bases** | 8,055,265,060 | 8,872,968,938 |
