# Supplementary material for: Comparative Transcriptome Analysis of Isoetes Sinensis Under Terrestrial and Submerged Conditions
Source: Plant Mol Biol Report. 2015 Jun 27;34:136–45. doi: 10.1007/s11105-015-0906-6 (PMC4722078; doi:10.1007/s11105-015-0906-6)
Supplement: Supplementary file 7 — Summary of unigenes in this study. (DOCX 15 kb) [file 11105_2015_906_MOESM5_ESM.docx]

**Table S 3** Summary of unigenes in this study.

|  | Unigenes |
| --- | --- |
| Total length(bp) | 51,156,918 |
| Numbers | 31,619 |
| Max length(bp) | 16,900 |
| Average length(bp) | 1,618 |
| N50 | 2,350 |
| >N50 reads numbers | 7,368 |
| GC(%) | 41.68% |
