# Supplementary material for: Comparative Transcriptome Analysis of Isoetes Sinensis Under Terrestrial and Submerged Conditions
Source: Plant Mol Biol Report. 2015 Jun 27;34:136–45. doi: 10.1007/s11105-015-0906-6 (PMC4722078; doi:10.1007/s11105-015-0906-6)
Supplement: Supplementary file 10 — Statistical analysis of carbohydrate metabolism under terrestrial and submerged conditions. Seventy-nine DEGs are significantly enriched in “carbohydrate metabolism” with 28 up-regulated and 51 down-regulated genes. (DOCX 17 kb) [file 11105_2015_906_MOESM8_ESM.docx]

**Table S6**  Statistics analysis of carbohydrate metabolism under under terrestrial and submerged conditions.

79 DEGs are significantly enriched in the “carbohydrate metabolism” category with 28 up-regulated and 51 down-regulated genes.

| Carbohydrate Metabolism | Up-regulated | Down-regulated | Totals |
| --- | --- | --- | --- |
| Starch and sucrose | 7 | 8 | 15 |
| Glycolysis | 0 | 14 | 14 |
| Citrate cycle | 0 | 3 | 3 |
| Pentose phosphate | 0 | 3 | 3 |
| Pentose and glucuronate interconversions | 0 | 1 | 1 |
| Fructose and mannose | 1 | 5 | 6 |
| Galactose | 3 | 1 | 4 |
| Ascorbate and aldarate | 1 | 1 | 2 |
| Amino sugar and nucleotide sugar | 8 | 3 | 11 |
| Pyruvate | 1 | 8 | 9 |
| Glyoxylate and dicarboxylate | 3 | 1 | 4 |
| Propanoate | 0 | 1 | 1 |
| Butanoate | 1 | 0 | 1 |
| Inositol phosphate | 3 | 2 | 5 |
| Totals | 28 | 51 | 79 |
